# Enhance transgene responses through improving cellular uptake and intracellular trafficking by bio-inspired non-viral vectors

**DOI:** 10.1186/s12951-020-0582-z

**Published:** 2020-01-31

**Authors:** Xi-Xi Ma, Jing-Liang Xu, Yi-Yang Jia, Ya-Xuan Zhang, Wei Wang, Chen Li, Wei He, Si-Yuan Zhou, Bang-Le Zhang

**Affiliations:** 1 Department of Pharmaceutics, School of Pharmacy, Fourth Military Medical University, Xi’an, 710032 China; 2 Key Laboratory of Pharmacology of the State Administration of Traditional Chinese Medicine, Fourth Military Medical University, Xi’an, 710032 China; 3 Department of Chemistry, School of Pharmacy, Fourth Military Medical University, Xi’an, 710032 China

**Keywords:** Cellular uptake pathway, Intracellular trafficking, Non-viral vectors, Transgene

## Abstract

**Background:**

Gene therapy remains a significant challenge due to lots of barriers limiting the genetic manipulation technologies. As for non-viral delivery vectors, they often suffer insufficient performance due to inadequate cellular uptake and gene degradation in endosome or lysosome. The importance of overcoming these conserved intracellular barriers is increasing as the delivery of genetic cargo.

**Results:**

A surface-functionalized non-viral vector involving the biomimetic mannitol moiety is initiated, which can control the cellular uptake and promote the caveolae-mediated pathway and intracellular trafficking, thus avoiding acidic and enzymatic lysosomal degradation of loaded gene internalized by clathrin-mediated pathway. Different degrees of mannitol moiety are anchored onto the surface of the nanoparticles to form bio-inspired non-viral vectors and CaP-MA-40 exhibits remarkably high stability, negligible toxicity, and significantly enhanced transgene expression both in vitro and in vivo.

**Conclusions:**

This strategy highlights a paradigmatic approach to construct vectors that need precise intracellular delivery for innovative applications.

## Background

Gene therapy is a kind of biomedical treatment, showing a promising therapeutic prospect for inherited and acquired diseases, such as cancer, viral infection, diabetes and AIDS [1–7]. Given the easy preparation, high gene loading efficiency and low immunogenicity, non-viral delivery vectors have attracted considerable attention in the gene therapy compared with viral delivery vectors [1, 8, 9]. However, the poor intracellular bioavailability and rapid degradation of the gene in the blood circulation, endosome or lysosome hinder their clinical application. It is well known that the lack of safe and efficient non-viral delivery vectors seriously influences the therapeutic efficacy in the clinic [10, 11].

**Fig. 1:**
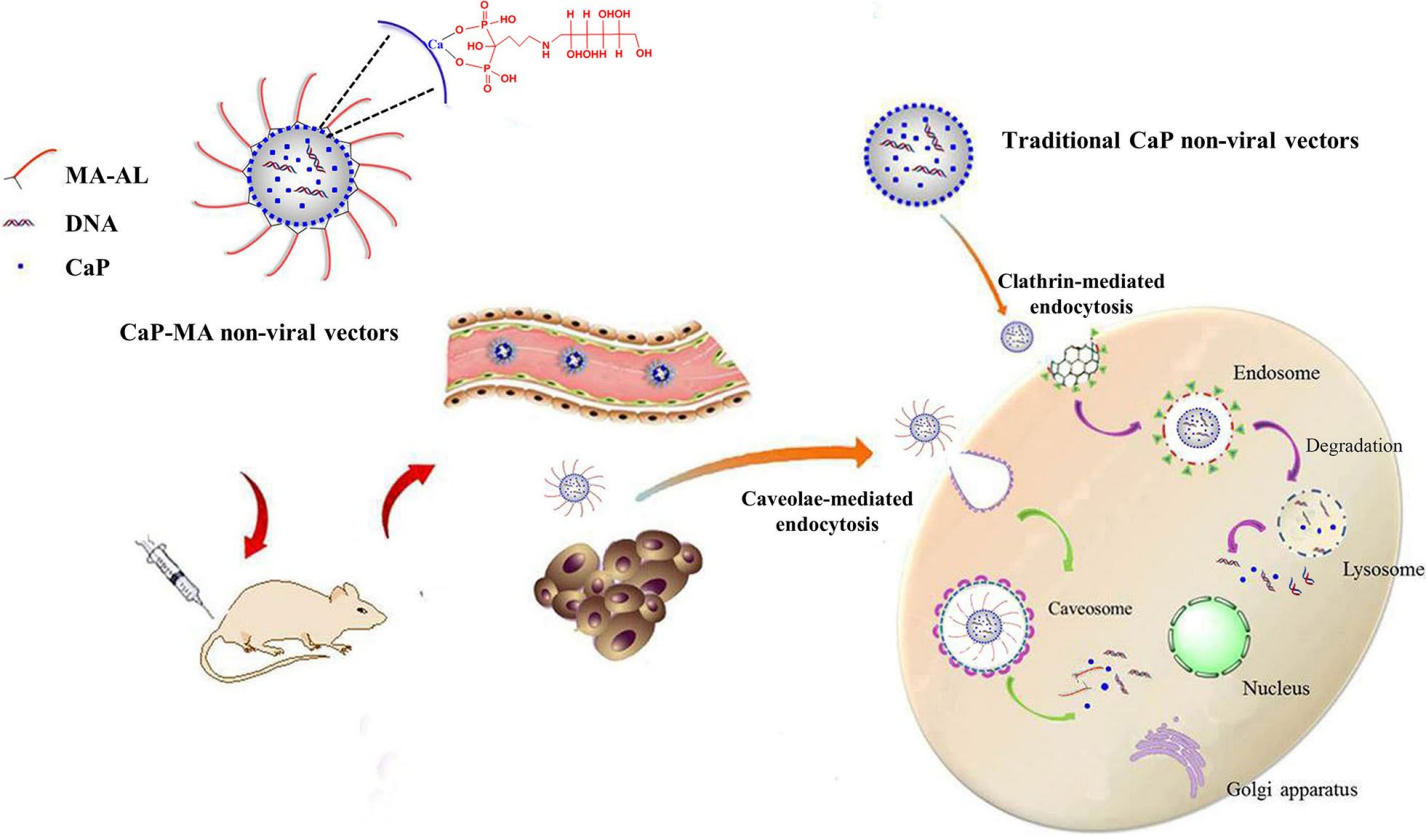
Schematic representation for the cellular uptake and intracellular trafficking of bio-inspired CaP-MA non-viral vectors

To date, numerous researchers focused on the design and construction of gene delivery vectors and made attempts to address the challenges. As for the non-viral delivery vectors, they often suffer insufficient performance due to poor transfection efficiency, relatively high toxicity, inadequate cellular uptake and gene degradation in endosome or lysosome, which significantly hampers the application in the clinic [1, 12–14]. Viral delivery vectors possess innate machinery to overcome cellular barriers, however, non-viral delivery vectors require great effort to rationally design to overcome these barriers. It has been confirmed that the cellular uptake pathways involved in traditional non-viral vectors include mainly the clathrin-mediated pathway, as well as the caveolae-mediated pathway [15–18]. Different uptake pathways result in totally different intracellular trafficking fates of delivery vectors. The endocytic vesicles internalized through the clathrin-mediated pathway are readily entrapped into endosome and then transfer their cargoes to lysosome followed by enzymatic degradation (Fig. 1) [19, 20]. On the contrary, the caveosome, endocytic vesicles of caveolae-mediated pathway budding from caveolae, does not lead to the degradative environment, thus avoiding the gene degradation in the lysosome [21–23]. Therefore, controlling the cellular uptake and consequent intracellular fates may be a promising paradigm to improve the transgene efficiency of traditional non-viral delivery vectors.

It has been testified that the external stimulating factors, such as hypoxia and hyperosmotic stress could modulate the function of caveolin and selectively stimulate and enhance the caveolae-mediated cellular uptake pathway [24–27]. Multi-hydroxyl compound mannitol has been commonly utilized as an organic osmolyte in the clinic [28–30], which inspires us to exploit unique, effective strategies to construct biomimetic non-viral vectors with controlled cellular uptake and consequent intracellular trafficking fates. Herein, through bio-inspired modification, a series of surface-functionalized non-viral vectors were constructed for the first time by introducing biomimetic moiety of mannitol-based mannitol-alendronate (MA-AL) to anchor onto the surface of nanoparticles (Fig. 1). Through coordination interaction between the phosphonate groups of MA-AL and the Ca^2+^ of nanoparticles, different degrees of MA-AL was anchored on the core of calcium phosphate (CaP) to self-assembly form the CaP-MA nanovectors. When loaded with DNA, the constructed non-viral vectors with mannitol groups may simulate caveolae-mediated cellular uptake, the non-destructive delivery pathway, to reduce the gene degradation in endosomes/lysosomes occurred with the clathrin-mediated pathway (Fig. 1). The endocytic uptake mechanism, intracellular trafficking fates, stability, cytotoxicity, and transgene expression in vitro and in vivo were investigated in details to demonstrate the favorable transgene responses.

## Results

### Preparation and characterization of the functionalized non-viral nanovectors

Reductive amination reaction was utilized to synthesize mannitol-alendronate (MA-AL). Aldehyde group of mannose reacted with the amino group of alendronate to form the Schiff-base and then sodium cyanoborohydride (NaBH_3_CN), the reductive agent, was added into the system leading to form the conjugated MA-AL. The synthetic route of MA-AL was illustrated in Additional file 1: Figure S1A. The structure of MA-AL was confirmed by ^1^H-NMR spectra and FTIR spectra (Additional file 1: Figure S1B, C). ^1^H-NMR (400 MHz, D_2_O): δ (ppm) 1.86–1.92 (m, 4H, H of alendronate), 2.92 (m, 2H, H of alendronate), 3.03–3.05 (m, 2H, H of mannitol), 3.45–3.89 (m, 12H, H of mannitol); IR (KBr): 3417, 3386, 1623, 1180, 1064, 690 cm^−1^. In this research, a facile co-precipitation method was employed to prepare DNA loaded nanoparticles. Then, through the coordination interaction between the phosphonate groups of the MA-AL and the Ca^2+^ of the core, MA-AL was anchored onto the surface of the CaP with different degrees to obtain the functionalized CaP-MA non-viral vectors. According to the concentrations of MA-AL (5, 20 and 40 μM) used in the preparation, the functionalized non-viral nanovectors were named CaP-MA-5, CaP-MA-20 and CaP-MA-40, respectively.

The particle size, zeta potential and stability of DNA loaded nanoparticles are pivotal properties for gene delivery, which affect the quality control and subsequent applications. Concerning size and stability, the samples were observed immediately after preparation and then after 24, 48, 72, 96 and 120 h, during which the nanoparticles were stored at 4 °C.

As shown in Additional file 1: Figure S1, during the determination by dynamic light scattering (DLS) assay, we found that the particle size of unanchored CaP nanoparticles had increased more than four times within 24 h. After 48 h, the intensity of the nanoparticle suspension was too low to be determined (Additional file 1: Figure S1D), showing poor colloidal stability because of the growth, aggregation and sedimentation of the nanoparticles. The zeta potential of the CaP nanoparticles was − 14.4 mV. When anchored with mannitol groups, the zeta potential of the CaP-MA-5/20/40 nanoparticles were − 16.4, − 19.4 and − 16.3 mV, respectively. The morphology images of scanning electron microscopy showed that both CaP nanoparticles and CaP-MA-5/20/40 nanoparticles had elongated or irregular aggregated micrographs in suspension with the particle sizes of about 650 nm for CaP nanoparticles and 350 nm for CaP-MA-5/20/40 nanoparticles (Additional file 1: Figure S1H), which is consistent with the size obtained by dynamic light scattering (DLS) assay. Considering the size measurement obtained by DLS and scanning electron microscopy, similar elongated aggregated structures were often observed on CaP nanoparticles in the previous reports [31, 32]. In addition, the size of CaP-MA nanoparticles was smaller than that of CaP nanoparticles and the stabilities of CaP-MA-20 and CaP-MA-40 were much better than those of CaP nanoparticles and CaP-MA-5 nanoparticles with low degree modification (Additional file 1: Figure S1), which indicated that the introduction of MA-AL was accompanied with enhanced stability and the stability was also associated with the degree of MA-AL used. The smaller size and remarkably enhanced colloidal stability were owe to the nano self-assembly stabilization of hydrophilic multi-hydroxyl groups on the surface of CaP-MA nanoparticles, which laid a solid foundation for clinical applications.

### DNA protection assay

The DNA protection experiment was carried out to assess the ability of CaP-MA non-viral vectors to protect nucleic acid under DNase I condition, simulating the environment in vivo. The results in Additional file 1: Figure S1H showed that CaP-MA nanoparticles have almost the same ability to protect DNA as the CaP nanoparticles. After incubating with DNase I, DNA in CaP and CaP-MA groups had no obvious change, while the naked DNA had been degraded by enzymolysis. In a word, all the three degrees functionalized CaP-MA non-viral vectors sustained the excellent capability of DNA protection as CaP nanoparticles.

### Evaluation of biocompatibility

Biocompatibility is crucial to gene delivery vectors, which influences their application in vitro and in vivo. It is well known that when using CaP nanoparticles alone, morphologic changes of cells were observed under the microscope during the transfection, and these changes can affect the cell status and result in relatively high cytotoxicity [33]. The toxicity of CaP and CaP-MA non-viral vectors were tested in HEK293T cells. Obviously, the biocompatibilities of CaP-MA groups were enhanced to a large extent compared with the unmodified CaP nanoparticles (Additional file 1: Figure S2A). At the concentration of 11.6 μg/mL used for transfection, even up to 40 μg/mL, the cytotoxicity was ignorable. Moreover, the cytotoxicity of MA-AL was also determined. The results showed that even in the case of the concentration of 800 μg/mL, 40 times higher than the modified degree of CaP-MA-40 non-viral vectors, the cytotoxicity still could be neglected (Additional file 1: Figure S2B). In addition, the hemolytic effect of CaP-MA nanoparticles was also determined and the results revealed that hemolytic rates of all modified nanoparticles were less than 5% (Additional file 1: Figure S2C, D), which met the requirements of safe materials [34]. Based on the results above, we concluded that all CaP-MA non-viral vectors had excellent biocompatibility and could be used as candidates for gene delivery.

### In vitro gene transfection

**Fig. 2:**
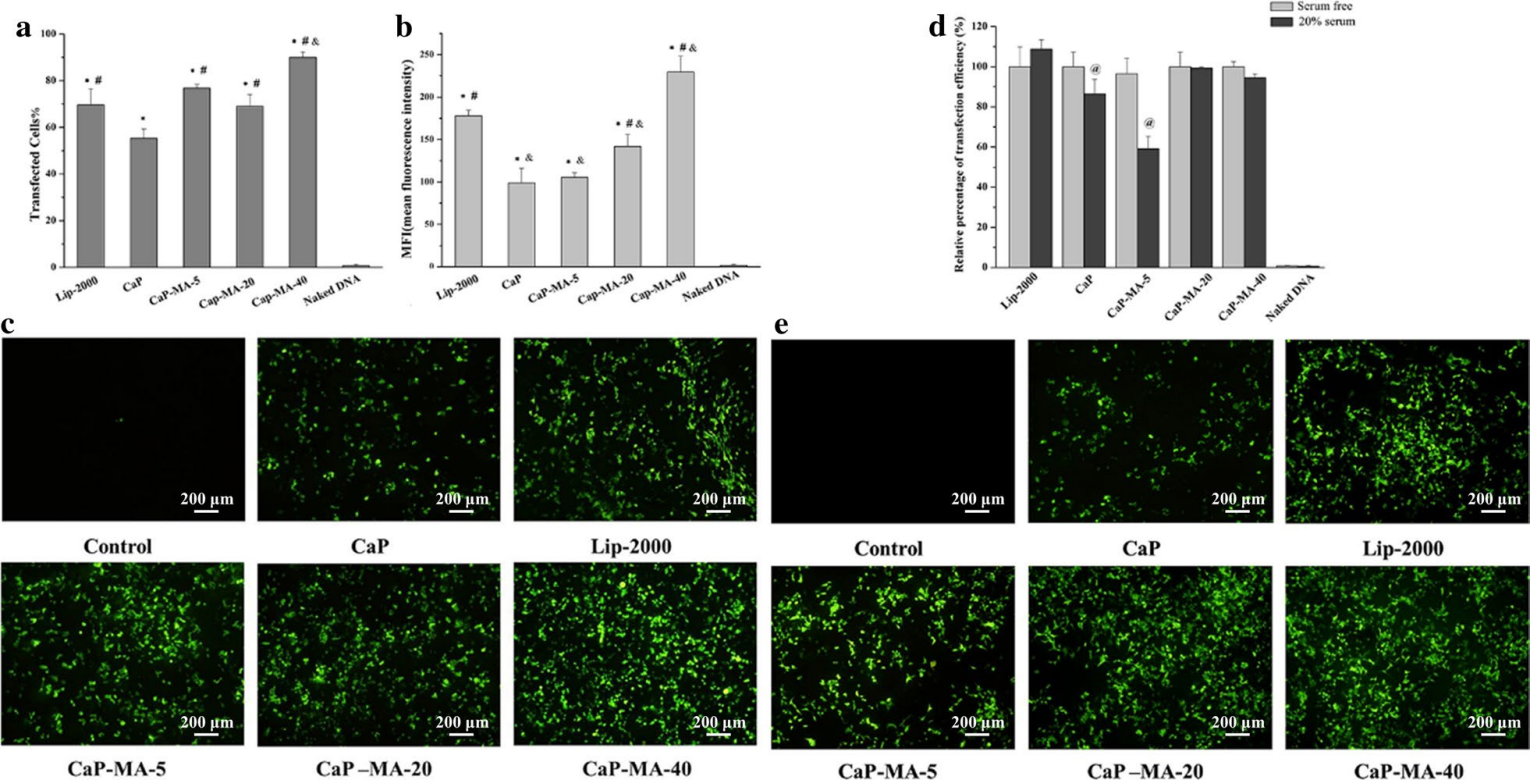
The percentage of positive transfected cells (transfected Cells%, **a**), mean fluorescent intensity (MFI, **b**) and green fluorescence protein expression (**c**) without serum; relative percentage of transfection efficiency (**d**) and green fluorescence protein expression in the presence of serum (**e**) observed by fluorescence microscopy (4×). Data are displayed as mean ± SD (n = 3). *P < 0.05, compared with naked DNA, ^#^P < 0.05, compared with CaP, ^&^P < 0.05, compared with Lipofectamine-2000 (Lip-2000), ^@^P < 0.05, compared with serum free group

The results presented in Fig. 2 indicated that all the modified CaP-MA non-viral vectors exhibited distinguished transgene expression and their transfection efficiency were all superior to CaP nanoparticles. Among them, the transfected cells% of CaP-MA-5 and CaP-MA-20 were comparable to Lip-2000, and CaP-MA-40 was better even than that of Lip-2000 (Fig. 2a). As for mean fluorescence intensity (MFI, Fig. 2b), CaP-MA-20 exhibited higher value than CaP, and CaP-MA-40 had the highest MFI among them including Lip-2000. Moreover, the images of fluorescence microscopy (Fig. 2c) were consistent with the results analyzed by FACS. Transgene experiments were also carried out in the presence of serum, and the relative percentage of transfected cells compared to their own serum free condition was shown in Fig. 2d, e. As for CaP and CaP-MA-5 nanoparticles with no or low degree of mannitol moieties, the gene transfection efficiency decreased significantly in the presence of serum. After anchored with mannitol groups, CaP-MA-20 and CaP-MA-40 with high degree of mannitol moieties could maintain their transfection activities in serum, having favorable serum compatibility for in vivo application. Taking stability, cytotoxicity, and transgene expression into consideration, CaP-MA-40 was selected as a potent transfection vector for gene delivery.

### Intracellular trafficking by laser scanning confocal microscopy

Cellular endocytic pathway and subsequently intracellular fates of gene delivery carriers are critical in gene transfection, which can influence the transfection activity remarkably [35]. In order to confirm the design in the present work and intuitively observe the intracellular trafficking fates of CaP and CaP-MA-40 non-viral vectors, the subcellular colocalization of nanoparticles was monitored by using specific molecular probes and fluorescein-labeled DNA nanoparticles in HEK293T cells. The molecular probes LysoTracker® Deep Red and Alexa Fluor 555 Cholera Toxin Subunit B (CT-B) Conjugates can selectively accumulate in lysosome and caveosome and trace clathrin-mediated pathway and caveolae-mediated pathway, respectively.

**Fig. 3:**
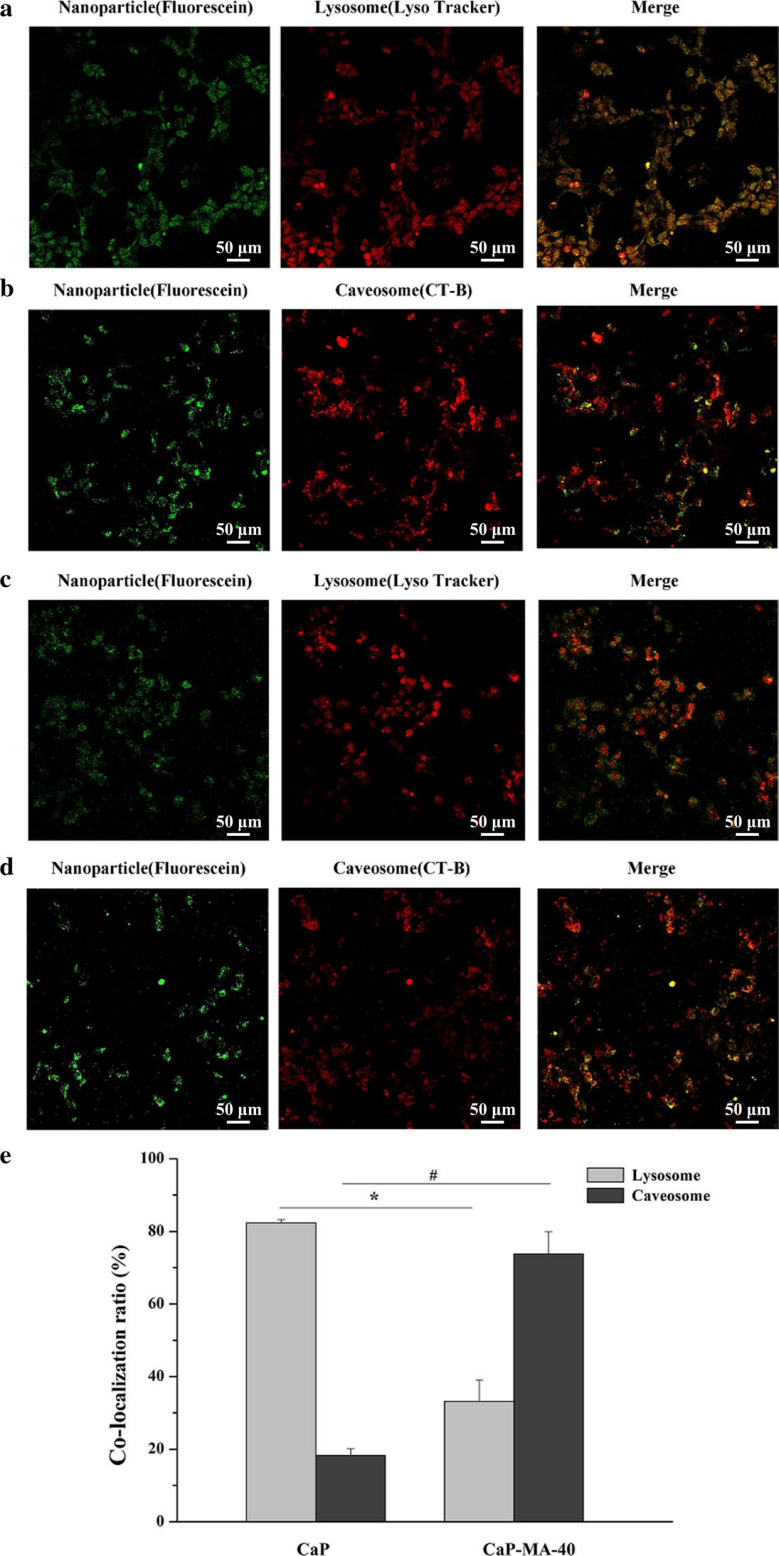
Subcellular colocalization of fluorescence-labeled CaP nanoparticles (**a**, **b**) and CaP-MA-40 nanoparticles (**c**, **d**) with molecular probes of lysosome or caveosome in HEK239T cells observed by Laser Scanning Confocal Microscopy. The colocalization ratio of nanoparticles with lysosomes or caveosome (**e**). LysoTracker® Deep Red and CT-B were used as specific molecular probes to label lysosome and caveosome, respectively. *P < 0.05, colocalization ratio of CaP with lysosome compared with the counterpart in CaP-MA-40 group, ^#^P < 0.05, colocalization rate of CaP with caveosome compared with the counterpart in CaP-MA-40 group

When cells were incubated with unmodified CaP nanoparticles and then marked by LysoTracker® Deep Red, the merged image exhibited an extremely high level overlap of subcellular colocalization in the lysosome (Fig. 3a). On the contrary, a much lower degree of overlap was observed in the colocalization image with the caveosome (Fig. 3b). These results indicated that the unmodified CaP nanoparticles were mainly internalized and transferred to lysosome (Fig. 3e). However, when cells were treated with mannitol-modified hybrid CaP-MA-40 nanoparticles, the degree of overlap in lysosome obviously reduced comparing with CaP (Fig. 3c), and fluorescence-labeled nanoparticles were mainly observed in caveosome (Fig. 3d, e). It is convinced that the introduction of MA-AL to CaP-MA-40 nanoparticles can transfer them into caveosome, avoiding the subsequent gene degradation in lysosome, which was of great importance in enhancing transgene activities.

### Endocytic mechanisms involved in CaP-MA non-viral vectors

**Fig. 4:**
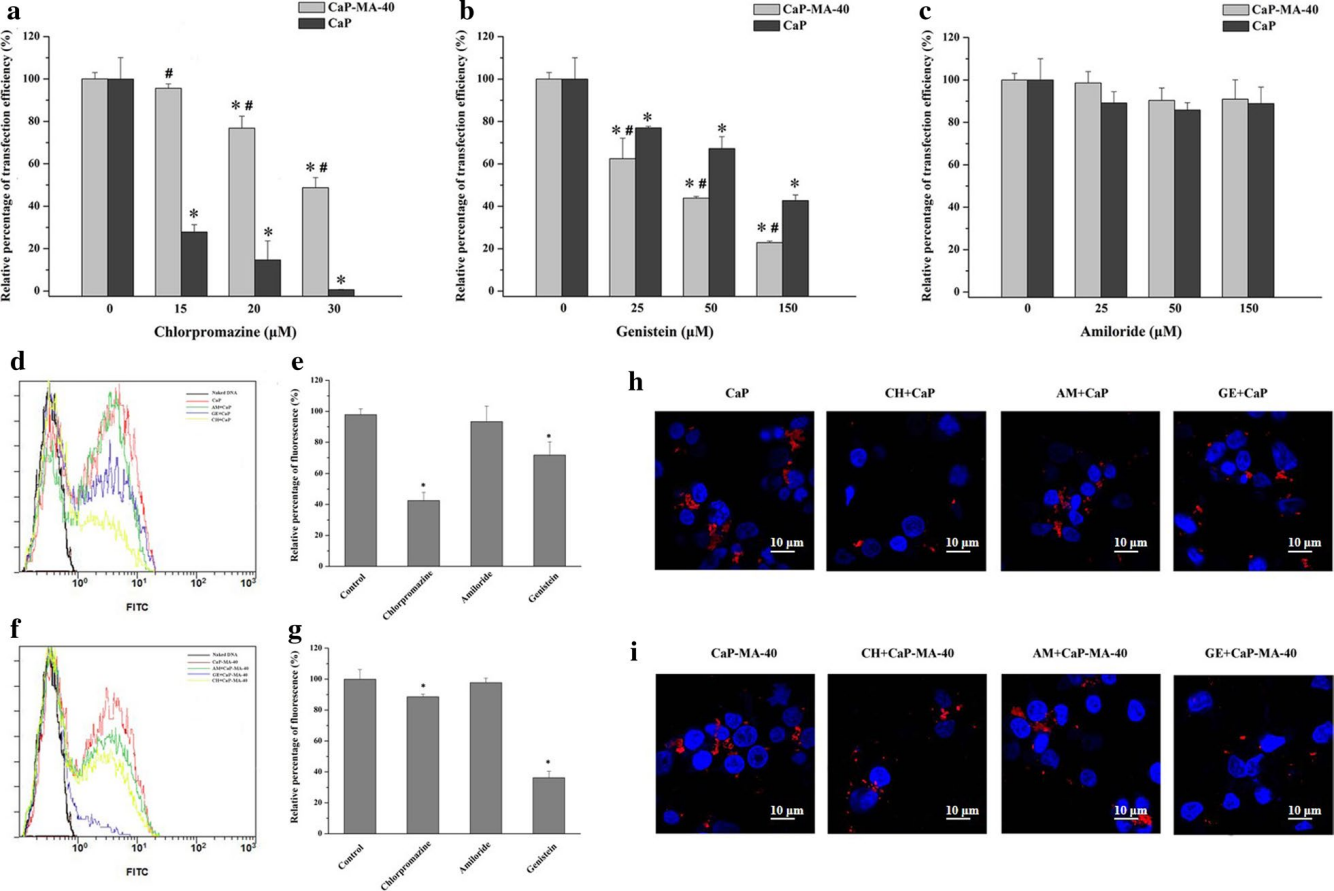
The influences on the transfection efficiency when treated with specific endocytic inhibitors amiloride (**a**), chlorpromazine (**b**) and genistein (**c**) and cellular uptake of fluorescence labeled CaP (**d**, **e**, **h**) and CaP-MA-40 (**f**, **g**, **i**) nanoparticles after treated with specific endocytic inhibitors in HEK293T cells. Data were represented as the relative transfection efficiency to the untreated cells group. Data are displayed as mean ± SD (n = 3). *P < 0.05, compared with the untreated cells group, ^#^P < 0.05, compared with the CaP group

To further reveal the mechanism of cellular uptake pathway, cellular uptake inhibitors were employed to investigate the differences in uptake behavior between unmodified CaP nanoparticles and CaP-MA nanoparticles. As the results shown in Fig. 4, when unmodified CaP nanoparticles were treated with chlorpromazine (CH), the inhibitor of clathrin-mediated uptake, at the concentration of 15, 20 and 30 μM, the transfection efficiency was reduced by 72.2%, 85.3% and 99.4%, respectively (Fig. 4a). And the decrease of transfection in the CaP group was markedly higher than that of multi-hydroxyl-modified CaP-MA-40 group at the corresponding concentration, while the CaP-MA-40 group decreased 4.4%, 23.2%, 51.3%, respectively. As to the caveolae-mediated uptake pathway (Fig. 4b), when cells were treated with genistein (GE), the transfection decrease in CaP-MA-40 group was significantly higher than that of CaP group. As displayed in Fig. 4b, the transfection efficiency of CaP-MA-40 was plunged to 37.6%, 56.2%, 77.2% at the concentration of 25, 50 and 150 μM, respectively, while CaP group decreased 23%, 32.8%, 57.3%, respectively. CaP-MA-40 nanoparticles had enhanced sensitivity to the inhibitor of genistein associated with the caveolae-mediated uptake pathway. When inhibited with amiloride (AM), the inhibitor of macropinocytic pathway, the transfection efficiency of CaP-MA-40 and CaP were unaffected even at the concentration up to 150 μM (Fig. 4c).

To ulteriorly investigate the correlation of the change of transfection with the cellular uptake, fluorescence labeled nanoparticles were utilized to quantitatively evaluate the uptake influences of specific inhibitors. As shown in Fig. 4d–i, the addition of amiloride did not impact the uptake both of unmodified CaP nanoparticles and CaP-MA-40 nanoparticles. When unmodified CaP nanoparticles were treated with the inhibitor chlorpromazine or genistein, the uptakes were inhibited 57.6% or 28.3%, respectively (Fig. 4d, e). As for CaP-MA-40 nanoparticles, the uptake reduced 11.5% or 63.8% (Fig. 4f, g), indicating that the mannitol-modified CaP-MA-40 nanoparticles had enhanced sensitivity to the inhibitor of genistein associated with the caveolae-mediated pathway. The cellular uptake images observed by laser scanning confocal microscopy were consistent with the results analyzed by FACS (Fig. 4h, i).

These results demonstrated that the endocytic uptake mechanisms involved in CaP nanoparticles were through the clathrin-mediated and caveolae-mediated cellular uptake pathway, but mainly in the clathrin-mediated pathway which subsequently delivered cargo into the endosome and then transferred into the lysosome, which led to DNA degradation and low transfection efficiency [13, 18, 19]. Thus, it was very crucial to alter the cellular uptake pathway and subsequently change the intracellular profiles for improving the efficacy of gene vectors. As the above results, the introduction of mannitol can alter the uptake pathway of CaP nanoparticles and a much stronger distinction in the change of uptake behavior between CaP and CaP-MA-40 was observed. The mannitol-modified CaP-MA-40 nanoparticles exhibited a reduced dependence on clathrin-mediated pathway (Fig. 4a, g) that trafficked to lysosomes and utilized caveolae-mediated pathway to a greater degree than CaP nanoparticles (Fig. 4b, e), which can reduce the pathway of gene degradation in endosome or lysosome and display high gene transfection efficiency, clearly suggesting a prominent role for the introduction of mannitol moiety in the uptake of CaP-MA-40 non-viral vectors.

### Western blot analysis of phosphorylated caveolin-1

To understand the mechanism of the observed change of the uptake pathway and the corresponding effects of introducing multi-hydroxyl mannitol moiety, a western blot experiment was performed. There have been reports elucidating that external stimulating factors could modulate the function of caveolin such as hypoxia and osmotic stress [25, 36, 37]. As a crucial type of caveolin, caveolin-1 (CAV1) is essential for caveolae formation and has serious effects on the function of cellular uptake [36, 38]. According to existed report, extracellular hyperosmotic stimulation could activate Src-kinase and further give rise to phosphorylation of CAV1, which plays a crucial part in the budding and releasing of cavelae in caveolae-mediated cellular uptake [26, 39].

**Fig. 5:**
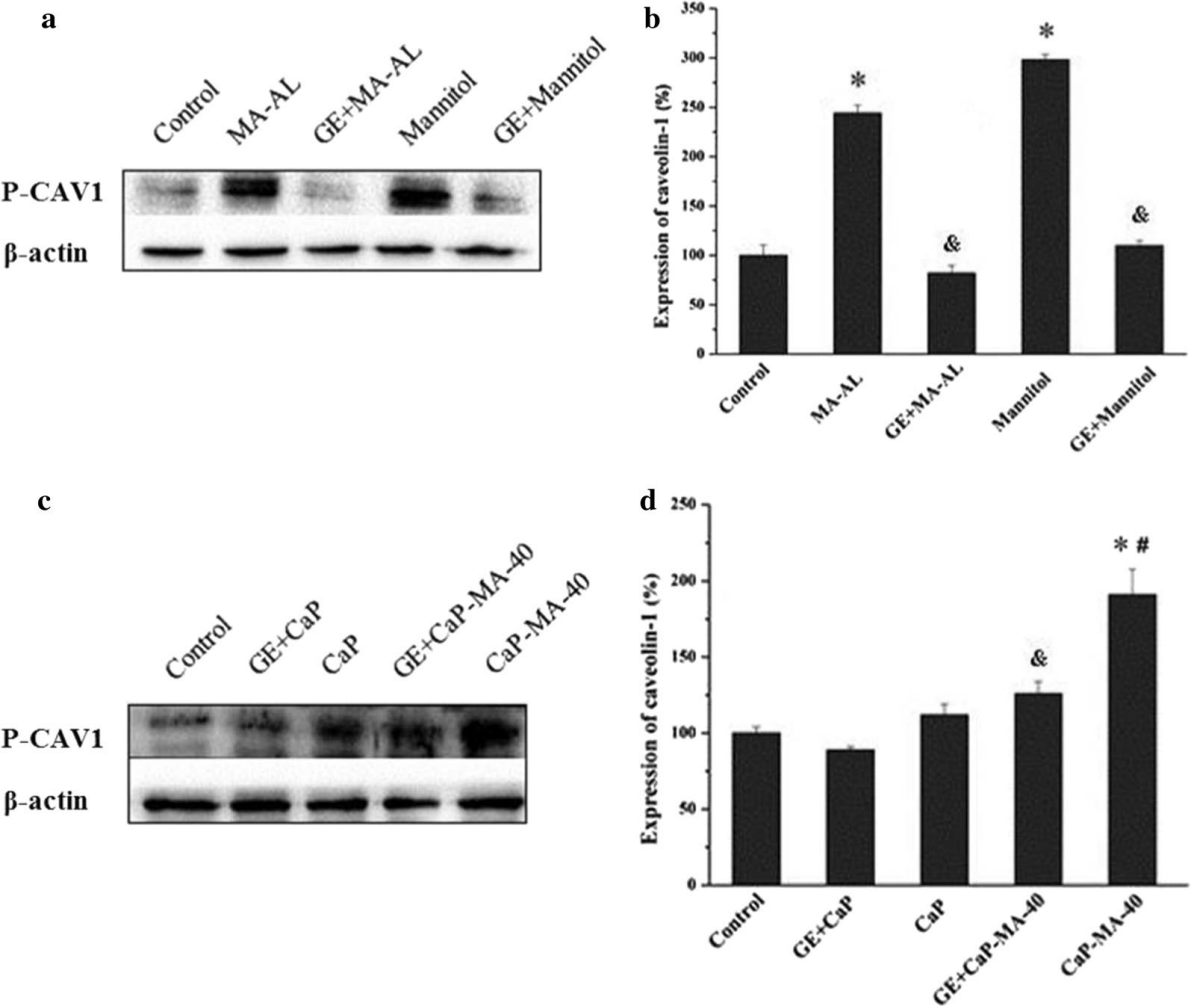
Evaluation of P-CAV1 expression by western blot experiment. **a**, **b** Cells were treated with Mannitol and MA-AL, respectively. **c**, **d** Cells were treated with CaP and CaP-MA-40 nanoparticles, respectively. GE + CaP, GE + CaP-MA-40, GE + Mannitol and GE + MA-AL groups were pre-incubated with genistein (GE) and untreated cells as the negative control, mean ± SD, n = 3. *P < 0.05, CaP, CaP-MA-40, Mannitol and MA-AL compared with control, ^#^P < 0.05, CaP-MA-40 compared with CaP, ^&^P < 0.05, GE + CaP-MA-40 compared with CaP-MA-40, GE + CaP compared with CaP, GE + Mannitol compared with Mannitol, and GE + MA-AL compared with MA-AL

As the results displayed in Fig. 5a, b, when cells were treated with mannitol and MA-AL, quantitative analysis exhibited accelerated expression of P-CAV1, which testified that the mannitol and MA-AL truly activated phosphorylation of CAV1, and stimulated the expression of P-CAV1. On the other hand, as for unmodified CaP nanoparticles (Fig. 5c, d), P-CAV1 did not change too much compared with the control because the caveolae-mediated pathway was not the main pathway in the uptake of traditional CaP nanoparticles, which was consistent with the results of cellular uptake test. When treated with mannitol-modified CaP-MA-40 nanoparticles, P-CAV1 up-regulated significantly (Fig. 5b), in other words, the phosphorylation of CAV1 took place during the uptake of CaP-MA-40 nanoparticles. Furthermore, when cells were pre-incubated with genistein (GE), specifically inhibiting the activity of Src tyrosine kinase and phosphorylation of CAV1 [40], the expression of P-CAV1 plugged into a low level. The decrease as shown in Fig. 5 signified that GE inhibited the phosphorylation of caveolin-1 activated by mannitol-modified CaP-MA-40 nanoparticles. Combined with the previous results, we were able to address the fundamental profile that mannitol moiety on the surface of CaP-MA-40 can induce the phosphorylation of caveolin-1 and activate the caveolae-mediated cellular uptake, thus altering the uptake pathway and intracellular trafficking of traditional nanoparticles.

### In vivo transgene experiments

**Fig. 6:**
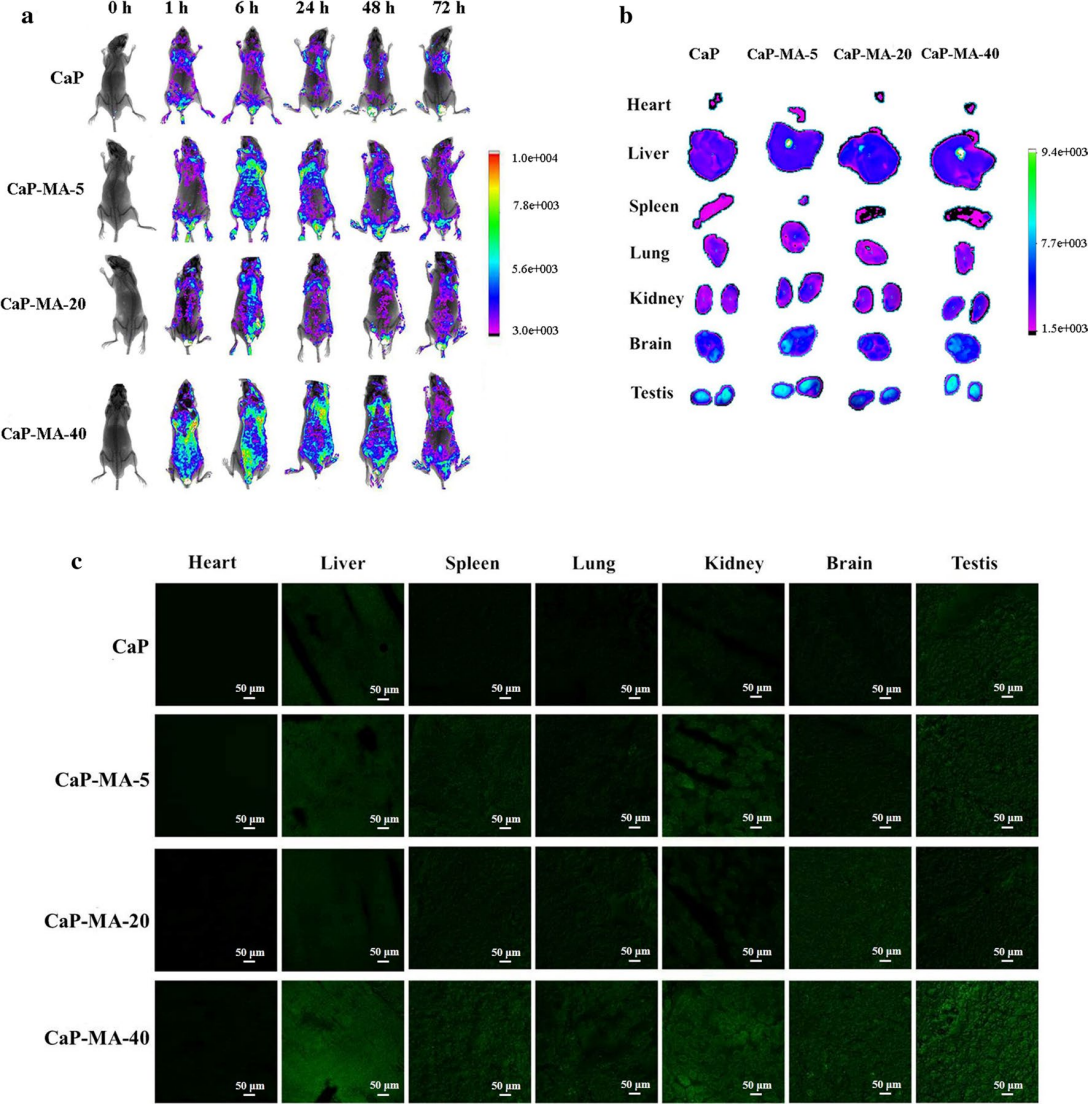
In vivo transgene efficiency evaluation performed on nude mice. **a** Fluorescence images of transfected nude mice at different time points after single tail-vein injection of CaP and CaP-MA nanoparticles containing EGFP plasmid. **b** Fluorescence images of excised organs at 6 h after tail-vein injection. **c** Frozen section of organs at 6 h after tail-vein injection observed by laser scanning confocal microscopy

After demonstrating efficient transgene with negligible cytotoxicity in vitro, the potential of mannitol-modified CaP-MA non-viral vectors in vivo were investigated. Although traditional CaP nanoparticles is facile to prepare, the significant variation of particle size elicits problems of reproducibility, potential risks and insufficient transgene performance in animal experiments [31–33]. Furthermore, the low transgene efficiency also hinders the application of CaP nanoparticles in vivo [12]. In this study, plasmids encoding enhanced green protein were used to observe the bio-distribution and transgene expression in vivo. After single dose administration, fluorescence imaging was performed at different time points including 1 h, 6 h, 24 h, 48 h and 72 h post-injection. As shown in Fig. 6a, one hour after injection, the GFP fluorescence can be detected, and CaP-MA-40 group exhibited the highest fluorescence imaging among four types of nanovectors, which is much better than CaP. The intensity of CaP-MA groups reached highest value at 6 h post-administration, and the GFP fluorescence could sustain at least 72 h in vivo after only single administration, achieving a long-term transgene expression. Moreover, the results also demonstrated that the transgene efficiency of the nanovectors were consistent with those of in vitro experiments.

To further investigate the distribution of transgene expression using different nanovectors, organs were harvested at 6 h post-administration. After being detected fluorescent intensities (MI) of excised organs (Fig. 6b), their frozen sections were also observed by confocal microscopy (Fig. 6c). The mean intensities of different organs ex vivo indicated that the MI of CaP-MA-40 was highest, in contrast to the lower fluorescence observed in organs harvested from other three groups. On the other hand, among the organs of CaP-MA-40 group, the descending sequence of MI was: testis (8055, 8408), liver (6611), brain (5253), kidney (4268, 4407), lung (3058), spleen (2219) and heart (1205). Surprisingly, the MI of testis was strongest among the organs and it was also universally acknowledged that the bio-distributions were associated with the potential application such as organs imaging and targeting [41–43]. Furthermore, the GFP expression of different organs were also observed by laser scanning confocal microscopy (Fig. 6c). When we analyzed the results above, it was generally accepted that CaP-MA-40 was an excellent delivery carrier with excellent stability, high transgene efficiency and good biocompatibility, which could be a promising candidate as delivery nanovector both in vitro and in vivo for further application.

## Discussion

Gene therapy remains a significant challenge due to lots of barriers limiting the additional genetic manipulation technologies. As for systemic gene delivery, most non-viral delivery vectors suffer insufficient performance due to poor stability, relatively high toxicity and drug degradation in endosome or lysosome, which significantly hampers the application in the clinic. Altering the endocytic pathway to decrease the acidic and enzymatic degradation may be a promising paradigm to construct controlled biomaterials for systemic gene delivery. Improving the material properties to control the cellular uptake and consequent intracellular fates is essential for enhancing the performance of gene delivery vectors.

By means of bio-inspired modification, an organic–inorganic hybrid nanovector using the biomimetic mannitol moiety to stimulate the biofunction of caveolin was constructed by anchoring mannitol-based mannitol-alendronate (MA-AL) onto the surface of nanoparticles. Through coordination interaction between the phosphonate groups of MA-AL and the Ca^2+^ of nanoparticles, different degrees of MA-AL was anchored on the core to self-assembly form the CaP-MA nanovectors. The constructed non-viral vectors with mannitol groups may simulate caveolae-mediated cellular uptake, the non-destructive delivery pathway, and transferred the genetic cargo into caveosome to avoid the subsequent gene degradation in lysosome, which is very important for enhancing transgene activities. The favorable transgene responses were demonstrated and among them, CaP-MA-40 exhibited remarkably high stability, negligible toxicity, and significantly enhanced transgene expression both in vitro and in vivo.

## Conclusions

In this report, the functionalized non-viral delivery vectors are developed via bio-inspired organic–inorganic hybrid strategy through coordination interaction to introduce the biomimetic moiety of mannitol, which can control cellular uptake and promote the caveolae-mediated uptake pathway and intracellular trafficking. This effective nanoplatform highlights a paradigmatic approach to construct biocompatible functional vectors with potential in biomedicine and can be extended in the future to develop entirely new classes of vectors that need precise intracellular delivery for innovative applications.

## Methods

### Materials and animals

Alendronate, d-(+)-mannose and sodium cyanoborohydride were brought from Aladdin Industrial Cooperation (Shanghai, China). Chlorpromazine hydrochloride was brought from Tokyo Chemical Industry Co., Ltd. Amiloride was purchased from Shanghai Haoyuan Chemexpress Co., Ltd. Genistein was purchased from Sun Chemical Technology (Shanghai) Co., Ltd. 3-(4,5-Dimethylthiazol-2-yl)-2,5-diphenyl tetrazolium bromide (MTT) was purchased from Sigma (St. Louis, MO, USA). Label IT® Nucleic Acid Labeling Kit, Fluorescein was brought from Mirus Bio LLC (USA). Alexa Fluor 555 Cholera Toxin Subunit B (CT-B) Conjugates and LysoTracker® Deep Red were purchased from Molecular Probes, Inc (USA). Dulbelcco's modified Eagle culture medium (DMEM) and fetal bovine serum (FBS) were purchased from Gibco (Carlsbad, CA, USA). pEGFP-N1, encoding the enhanced green fluorescence protein (EGFP) under a CMV promoter, was bought from Shanghai GenePharma Co., Ltd. (Shanghai, China). The anti-caveolin-1(phospho Y14) antibody was brought from Abcam Company. Nude mice were purchased from Hunan SJA Laboratory Animal Co., Ltd and all animal experiments were complied with the regulations of Animal Ethics Committee of Fourth Military Medical University.

### Synthesis and characterization of MA-AL

Briefly, alendronate (1.62 g, 0.5 mmol) was dissolved in the 35 mL mixed solution of MeOH:H_2_O (1:2). The mixture was heated to 80 °C and stirred for 15 min. After alendronate being dissolved completely, mannose (1.17 g, 0.65 mmol) and sodium cyanoborohydride were added into the mixture. The reaction solution was stirred at 80 °C and monitored by thin layer chromatography (TLC). After being stirred for 72 h, the mixture was evaporated to remove MeOH and then filtered. With stirring, the filtrate was dialyzed rapidly with a dialysis tube (MWCO 100, Spectrumlabs, China) and the solution was collected and lyophilized for dryness to afford a white solid MA-AL. The structure of MA-AL was confirmed by ^1^H-NMR spectra in D_2_O (400 MHz, INOVA-400 MHz spectrometer) and Fourier transform infrared spectra (FTIR, FTIR-8400S spectrometer).

### Preparation of CaP-MA nanoparticles

2.5 M CaCl_2_ solution was mixed with 0.5 μg/μL pEGFP-N1 dissolved in DNase/RNase-Free ddH_2_O at a volume ratio of 1:1, and the mixture was incubated for 15 min at room temperature. After incubating, the pEGFP-N1/CaCl_2_ mixture was diluted five folds by ddH_2_O. During bubbling, the mixture was added into the same volume of 2 × HEPES buffer (0.05 M 4-(2-hydroxyethyl)-1-piperazineethanesulfonic acid, 0.28 M NaCl, 1.5 mM Na_2_HPO_4_, pH 7.05), and then MA-AL solution with the concentrations of 105 μM, 410 μM and 820 μM was mixed immediately at a volume ratio of 1:20 to form the CaP-MA nanoparticles. The final MA-AL concentrations used in the CaP-MA nanoparticles were 5, 20 and 40 μM, so we named them CaP-MA-5, CaP-MA-20 and CaP-MA-40, respectively.

### Characterization of CaP-MA nanoparticles

Three samples were prepared for each nanoparticle as description and particle size and zeta potential were measured by Dynamic Light Scattering (DLS) (Beckman Coulter, CA, USA) at 25 °C. Scanning electron microscopy (SEM) was carried out by HITACHI S-4800 (Hitachi, Japan). The stability experiments of CaP and CaP-MA nanoparticles were also analyzed by DLS, during which the nanoparticles were stored at 4 °C.

### DNA protection assay

Briefly, nanoparticles were prepared as described above, one half nanoparticle samples (containing 1 μg of plasmid) were incubated with DNase I (0.125 U/μg DNA) for 30 min. Then, 5 μL of EDTA solution (250 mM) was utilized to end the enzymatic digestion reaction. All samples were treated with 1% SDS, and then loaded onto a 0.7% agarose gel containing Gel Signal™ Green running at 90 V for 45 min. DNA was visualized on a UV transilluminator (Clinx ChemiScope 6300).

### Cell lines and cell culture

HEK293T cells (human embryonic kidney cells) were provided by the Culture Collection of the Chinese Academy of Science (Shanghai, China). Cells were maintained in the complete medium which contains DMEM with GlutaMAX supplemented, 10% FBS and 1% antibiotics (penicillin/streptomycin) in a 5% CO_2_ atmosphere at 37 °C. Subcultivation was done two or three times a week.

### Cell viability assay

The toxicity of nanoparticles or compounds toward HEK293T cells was assessed by the MTT assay. 20,000 cells were seeded into a 96-well plate and maintained in DMEM supplemented with 10% FBS at 37 °C for 24 h. When the original culture media were replaced by 100 μL of DMEM, nanoparticles or compounds in different concentrations were added. After 24 h incubation with nanoparticles, the original media was removed and washed twice with PBS. After that, 20 μL MTT solution (0.5 mg/mL) and 100 μL PBS were added and incubated for another 4 h. Then 150 μL of DMSO was added into per well to completely dissolved the reduced crystal violet through vortexing for 5 min. Finally, the absorbance value at 490 nm was recorded by the plate reader.

### Hemolysis experiment

Fresh whole blood samples were collected from rats and sodium heparin was added to anti-coagulate. After being diluted to 50% with normal saline, 200 μL of blood samples were incubated with CaP, CaP-MA-5, CaP-MA-20, CaP-MA-40 or Lip-2000 respectively for 1 h at 37 °C. Normal saline was employed as the negative control (blank), and 1% Triton-X was employed as the positive control (triton). The final concentration was the same as that used in the transfection experiment. Then the mixtures were centrifuged for 10 min at 2000 rpm and supernatants were collected. After being diluted to 50% with the mixture of ethanol and hydrochloric acid (v/v = 39/1), the absorbance of supernatants was detected at 398 nm by UV–visible spectrophotometry. The hemolytic rates were calculated as follow:

1 $${\text{Hemolytic rate }} \% = [({\text{nanoparticles{-}blank}})/({\text{triton{-}blank}})] \quad \times 100$$

## Gene transfection

HEK293T cells were seeded in 6-well plates at the concentration of 2 × 10^5^ cells per well in DMEM containing 10% FBS and incubated for 24 h. The culture medium was removed and replaced by 1 mL of DMEM. The nanoparticles with different modifications were prepared as description, and then 100 μL solution of nanoparticles was added to the medium (2.5 μg of DNA per well). After 8 h incubation, the media were removed, and then 2 mL of complete culture medium was added respectively. Plates were further incubated for 40 h, and the transfection was stopped. After trypsinizing and re-suspending in PBS (pH 7.4), triplicate samples were analyzed by flow cytometry (Becton and Dickinson flow cytometer). The data were calculated from three or four repetitions.

### Observation of fluorescent microscopy

To observe the fluorescence of GFP expression, cells were maintained in 24-well plates on coverslip at the density of 50,000 cells per well, and incubated for 24 h. For transfection, the nanoparticles were prepared as described and then added to the medium (0.8 μg of pDNA per well). After 8 h of incubation, the original medium was substituted by 500 μL complete culture medium. The transfection was finished after incubation for 40 h and fluorescence microscopy was employed to detect GFP expression.

### Determination of the cellular uptake pathway

The specific cellular uptake inhibitors were utilized to determine the endocytic pathways. The HEK293T cells were seeded in 6-well plates at the concentration of 2 × 10^5^ cells per well in DMEM containing 10% FBS and incubated for 24 h. Before the nanoparticles were added, cells were incubated with clathrin-mediated endocytic inhibitor chlorpromazine (15, 20 and 30 μM), caveolae-mediated endocytic inhibitor genistein (25, 50 and 150 μM), or macropinocytosis inhibitor amiloride (25, 50 and 150 μM) for 1 h. The nanoparticles with different modifications were prepared as described above, and then 100 μL solution of nanoparticles was added to the medium (2.5 μg of DNA per well) in the presence of the inhibitors. The following steps were the same as the transfection experiment in vitro mentioned above. For the control group, cells were not treated with the inhibitors. The results were expressed as % transfected cells compared with the control group without inhibitors.

### Determination of the cellular uptake

The HEK293T cells were seeded in 24-well plates and incubated for 24 h. Fluorescein was employed to label pDNA following the manufacturer's instructions. The corresponding CaP or CaP-MA-40 nanoparticles were prepared as described before. When the cells were incubated with chlorpromazine (30 μM), genistein (150 μM), or amiloride (150 μM) for one hour, the nanoparticles were added into the well (0.8 μg of pDNA per well) and further incubated for 4 h. After washing three times with PBS (pH 7.4), the cells were trypsinized and resuspended in PBS. Triplicate samples were analyzed by flow cytometry (Becton and Dickinson flow cytometer). To observe the uptake, cells were seeded in 24-well plates on the coverslip and other treatments were the same as described above. The samples were observed by laser scanning confocal microscopy (Olympus Fluoview FV1000).

### Intracellular trafficking fates by laser scanning confocal microscopy

The intracellular behavior of nanoparticles was monitored by subcellular colocalization with specific molecular probes. Fluorescein was employed to label pDNA following the manufacturer's instructions. Alexa Fluor 555 Cholera Toxin Subunit B (CT-B) Conjugates (4 μg/mL) and LysoTracker® Deep Red (50 nM) were utilized to trace caveolae-mediated uptake pathway and clathrin-mediated uptake pathway, respectively. HEK293T cells were seeded in 24-well plate on coverslips 24 h. The cells were incubated with fluorescein-labeled CaP or CaP-MA nanoparticles for 4 h and 500 μL of CT-B or LysoTracker® Deep Red solution were added and incubated at 37 °C for 40 min. After incubation, the cells were washed twice with PBS and fixed with paraformaldehyde. All the slides were visualized by laser scanning confocal microscopy.

### Western blot analysis

The protein expression of phosphorylated caveolin-1(P-CAV1) was analyzed by Western blot. HEK293T cells were seeded in 6-well plates at the concentration of 4 × 10^5^ cells in DMEM containing 10% FBS and incubated for 24 h. CaP and CaP-MA-40 nanoparticle was added respectively to the cells at the same concentration as transfection and incubated for 30 min with or without genistein. When treated with the inhibitor genistein (150 μM), it was added one hour before the incubation. The media were removed and the total protein of the cells was extracted by the method of RIPA, and the protein concentrations were detected by the BCA (Thermo, USA) methods. Equal amounts of protein were fractionated using a 15% SDS-PAGE gel and then transferred to a PVDF membrane (Millipore, USA), and then pre-blocked with 5% bovine serum albumin in Tris-buffered saline Tween 20 solution for 3 h at 37 °C. Primary antibodies of P-CAV1 (1:2500) and β-actin (1:5000) were diluted with a blocking solution containing 5% bovine serum albumin in TBST-2% Tween 20 overnight at 4 °C. After being washed with TBST-2% Tween 20, the blots were incubated at 37 °C for 2 h with horseradish peroxidase (HRP)-conjugated goat anti-rabbit IgG (1:5000 dilution). After being washed three times, the blots were visualized and the relative densities of the bands were quantified by Image J software. To confirm the hypertonic effects, 600 mM mannitol and MA-AL was used as the positive control.

### In vivo transgene experiments

The animal experiments were performed on six-week-old male nude mice, which were raised in the specific pathogen free house. The mice were divided into four groups randomly and 200 μL of CaP or CaP-MA-5/20/40 containing 10 μg DNA were injected intravenously via the tail vein. The normal saline was used as control. After injection for 1 h, 6 h, 24 h, 48 h, 72 h, the mice were anesthetized by isoflurane and the fluorescence observed by Xtreme BI system (Bruker USA). For fluorescence images of excised organs, 6 h after in vivo transgene, one mouse per group was shaved from the neck and fresh organs including spleen, liver, heart, lung, kidney, brain and testis were harvested and analyzed for the transgene expression using the Xtreme BI system (Bruker USA). After imaging, organs were then embedded in Tissue-Tek® opti-mum cutting temperature for frozen section and observed by laser scanning confocal microscopy.

### Statistical analysis

Data in the paper were expressed as mean ± SD. Statistical analysis was evaluated by One-Way ANOVA in SPSS 17.0. P < 0.05 was considered significant.

## Supplementary information

**Additional file 1: Figure S1.** Characterization of the functionalized CaP-MA nanoparticles. Synthetic route (A) and 1H NMR (B) and FTIR (C) spectrum of the conjugated mannitol-alendronate (MA-AL); Size distribution and polydispersity index (PDI) of CaP (D), CaP-MA-5 (E), CaP-MA-20 (F) and CaP-MA-40 (G); Morphology of CaP and different CaP-MA nanoparticles (H); Evaluation the abilities of different nanoparticles to protect DNA (I), (+) represented incubation with DNase I and (-) represented incubation without DNase I, mean ± SD, n=3. ★The intensity was too low to be determined. **Figure S2.** The biocompatibility of CaP and CaP-MA-5/20/40 nanoparticles. Cell viability of CaP nanoparticles, CaP-MA nanoparticles (A), and MA-AL (B) measured by the MTT assay. The mixture of fresh blood and nanoparticles after 1 hour incubation (C), Lip-2000 (a), CaP-MA-40 (b), CaP-MA-20 (c), CaP-MA-5 (d), CaP (e), saline (f), Triton-X (g). Hemolytic rates of Lip-2000, CaP and CaP-MA-5/20/40 nanoparticles (D). Data are shown as mean ± SD (n=3). * P<0.05, compared with the CaP group at the same concentration.

## Data Availability

All data generated or analyzed during this study are included in this published article.
