# Supplementary material for: Enhance transgene responses through improving cellular uptake and intracellular trafficking by bio-inspired non-viral vectors
Source: J Nanobiotechnology. 2020 Jan 31;18:26. doi: 10.1186/s12951-020-0582-z (PMC6995230; doi:10.1186/s12951-020-0582-z)
Supplement: Supplementary file 1 — Additional file 1: Figure S1. Characterization of the functionalized CaP-MA nanoparticles. Synthetic route (A) and 1H NMR (B) and FTIR (C) spectrum of the conjugated mannitol-alendronate (MA-AL); Size distribution and polydispersity index (PDI) of CaP (D), CaP-MA-5 (E), CaP-MA-20 (F) and CaP-MA-40 (G); Morphology of CaP and different CaP-MA nanoparticles (H); Evaluation the abilities of different nanoparticles to protect DNA (I), (+) represented incubation with DNase I and (-) represented incubation without DNase I, mean ± SD, n=3. ★The intensity was too low to be determined. Figure S2. The biocompatibility of CaP and CaP-MA-5/20/40 nanoparticles. Cell viability of CaP nanoparticles, CaP-MA nanoparticles (A), and MA-AL (B) measured by the MTT assay. The mixture of fresh blood and nanoparticles after 1 hour incubation (C), Lip-2000 (a), CaP-MA-40 (b), CaP-MA-20 (c), CaP-MA-5 (d), CaP (e), saline (f), Triton-X (g). Hemolytic rates of Lip-2000, CaP and CaP-MA-5/20/40 nanoparticles (D). Data are shown as mean ± SD (n=3). * P<0.05, compared with the CaP group at the same concentration. [file 12951_2020_582_MOESM1_ESM.doc]

**Additional file**

**Enhance transgene responses through improving cellular uptake and intracellular trafficking by bio-inspired non-viral vectors**

Xi-Xi Ma^1, 2^, Jing-Liang Xu^1, 2^, Yi-Yang Jia^1, 2^, Ya-Xuan Zhang^1, 2^, Wei Wang^1, 2^, Chen Li^2^, Wei He^3, *^, Si-Yuan Zhou^1, 2^ and Bang-Le Zhang^1, 2, *^

^1^ Department of Pharmaceutics, School of Pharmacy, Fourth Military Medical University, Xi’an, 710032, China; ^2^ Key Laboratory of Pharmacology of the State Administration of Traditional Chinese Medicine, Fourth Military Medical University, Xi’an, 710032, China; ^3^ Department of Chemistry, School of Pharmacy, Fourth Military Medical University, Xi’an, 710032, China

^*^Correspondence: blezhang@fmmu.edu.cn (BL Zhang); weihechem@fmmu.edu.cn (W He)


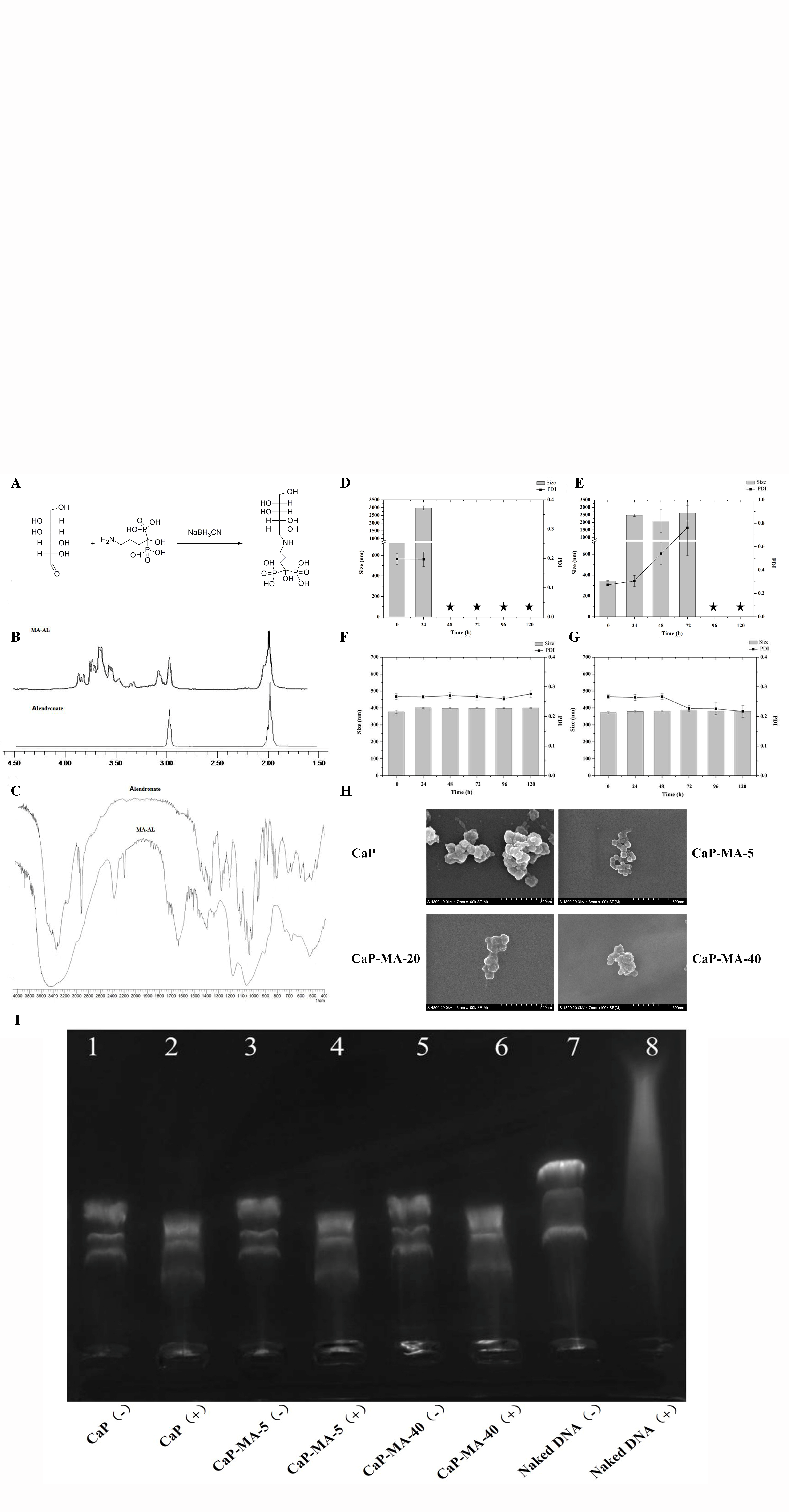


**Figure S1** Characterization of the functionalized CaP-MA nanoparticles. Synthetic route (A) and ^1^H NMR (B) and FTIR (C) spectrum of the conjugated mannitol-alendronate (MA-AL); Size distribution and polydispersity index (PDI) of CaP (D), CaP-MA-5 (E), CaP-MA-20 (F) and CaP-MA-40 (G); Morphology of CaP and different CaP-MA nanoparticles (H); Evaluation the abilities of different nanoparticles to protect DNA (I), (+) represented incubation with DNase I and (-) represented incubation without DNase I, mean ± SD, n=3. ^★^The intensity was too low to be determined.


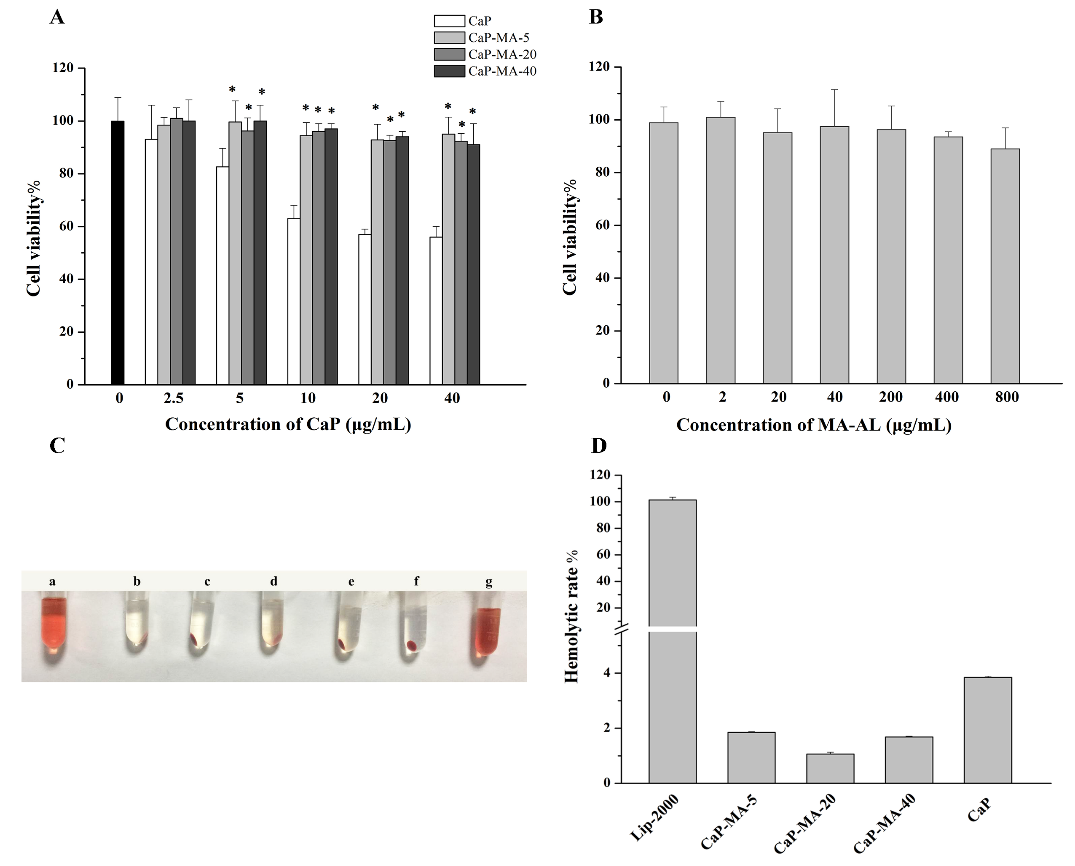


**Figure S2** The biocompatibility of CaP and CaP-MA-5/20/40 nanoparticles. Cell viability of CaP nanoparticles, CaP-MA nanoparticles (A), and MA-AL (B) measured by the MTT assay. The mixture of fresh blood and nanoparticles after 1 h incubation (C), Lip-2000 (a), CaP-MA-40 (b), CaP-MA-20 (c), CaP-MA-5 (d), CaP (e), saline (f), Triton-X (g). Hemolytic rates of Lip-2000, CaP and CaP-MA-5/20/40 nanoparticles (D). Data are shown as mean ± SD (n=3). ^*^ P<0.05, compared with the CaP group at the same concentration.
